# Our Dental Literature

**Published:** 1888-09

**Authors:** L. Ashley Faught


### OUR DENTAL LITERATURE.

BY L. ASHLEY FAUGHT, D. D. S.

Read before the Pennsylvania State Society, June 6, 1888.

Upon the cover of one of our journals we find these words: "Observe, compare, reflect, record." A glorious and effective motto! How has it been heeded by the profession?

The statistics I have to present shall be the evidence, and you, yourselves, shall be the judges.

For several years I have followed and compared the writers and writings found in the dental journals, to note the progress of dentistry. In my studies of the contributions there found, it has seemed to me that chaff exceeded wheat, and echoes exceeded voices.

I admit that a repetition of old ideas is needed, for some men require to have a new idea stated to them about twenty times before it is registered in their sensorial consciousness, and I sincerely believe that an article full of originality is seldom grasped in its entirety on its first publication. I would, however, advocate the relegation of repetition to the lecture desks of colleges or to the covers of adequately prepared text-books.

The students of the profession should be trained by our present college chairs, or by the establishment of a new one—that of dental literature—in all that has been presented to the profession, by all writings up to date of graduation. It should be the work of the professors or professor thus to send them forth fully acquainted with the past, and fully prepared to comprehend and contribute to the writings of the future. Journals, then, need contain only that which is new, since their last, and up to their immediate date of issue. Under this condition of affairs, practitioners would eagerly look for them, and turning their pages over and over in study, would thus keep upon the crest of the wave and ever be ready for progressive work. The occupants of professional positions, securing this material as it in turn became old, would incorporate it into their lectures to the coming recruits.

In order that this retrospect may not become too voluminous, I will confine my observations to the *Dental Cosmos,* as a typal journal, and to the years 1872 to 1887, inclusive.

During the last seven of these years, the character of the material presented has improved in scientific quality. This is not a mere impression, but a truth arrived at by careful comparison. Because of this marked contrast and for other satisfactory reasons, I shall present my statistics in two divisions, covering periods of nine and of seven years, respectively.

After carefully enumerating and tabulating, I have discovered that of the twelve thousand dentists in the United States, only three hundred and thirty-four, in those nine years, have contributed anything to its pages; and even this small number is attainable only by including every member reported as having made remarks in any dental society!—though he may only have said, "Those are my sentiments," or "I agree with the previous speaker."

Twenty-five of the contributors did more than one-third of the work, and fifteen of these occupied positions as teachers in dental institutions. From such we naturally expect to hear, as they are supposed to be gathering material in their experimental work as instructors. Can it be possible that there were only ten active minds in the private ranks of the profession of dentistry? And yet it seems but reasonable to infer that workers will desire the widest promulgation of their labors.

Making an exception of two serials, which were copyrighted for publication afterward, as text-books, I have also found that the number of subjects thus presented during this time is one hundred and thirteen, which may be classified as follows: Theoretical, sixty-eight; relating to materials and drugs, twenty-five; manipulation, fourteen; pathological and therapeutical, six. By this classification it will be seen that the bulk of the matter was theoretical, and which, even if handled in a masterly manner, is, at best, of questionable value.

The subjects receiving the most attention were: "Sixth-year Molars," "Eclectic Dentistry," "Neuralgia," "Caries," "Conservatism," "Doctor," "Pulpless Teeth," "Exposed Pulps," "Sensitive Dentine," "Contour Filling," "Creosote," and "Amalgams."

Let us consider the second period, from 1881 to 1887, inclusive. This should claim our especial interest, as it covers a period intimately connected with the present.

Counting in the same way as before—including all who gave the sound of their voice at a reported dental meeting—the number contributing during these seven years is two hundred and ninety-seven. Of these, thirty-two have done more than one-third of the work, nine being in professorial positions. The *active* workers, quoted in the *Cosmos* during this time in the *private* ranks, were only twenty-three.

The number of subjects presented during these seven years is one hundred and two, and may be classified as follows: Theoretical, forty-two; relating to materials and drugs, fourteen; manipulation, fourteen; pathological and therapeutic, thirty-two. The subjects receiving the most attention, stated in this instance, were: "Crown and Bridge-Work," "Dental Caries," "Regulating Teeth," "Filling Teeth," "Pulpless Teeth," "Dental Literature and Education," "Amalgam," and "Prosthesis."

Comparison between the latter seven years and the former nine shows a slight increase in the *general* workers, and a marked increase in the *active* workers in the private ranks. This has of course been naturally accompanied by an increase in the number of subjects treated, and their nature has also changed.

Theory still leads the list, though the proportion of interest in it is slightly decreased. Materials and drugs and manipulation receive about the same attention; while the trend of pathology and therapeusis is decidedly towards an advanced position.

The most marvelous thing, however, revealed by the analysis of the past seven years is the fact that the profession, as represented by its writers, has devoted the greater proportion of its powers to the study, development and consideration of crown and bridge-work. This subject has had prominence and preferment above every other subject. The discovery of this fact gave your essayist much food for meditation, as it probably will many of you; for, in his opinion, the profession has devoted too much valuable time to what is not calculated to be of general, every-day practical benefit; but adapted to very special practice, and therefore rather limited in its usefulness.

It is a matter of every-day remark, by many supposed to be informed upon the subject, and whose opinion is of value, that a dentist to be truly scientific must be medically educated. They would lead us by their remarks to infer that the thinkers and writers of the profession were those who held the title M. D. in addition to D. D. S.

The facts, so far as I can obtain them, show that the thinkers and writers holding the two titles are but thirty per cent. of the whole number. I must admit, however, that as a rule the most scientific literary work has been done by this thirty per cent., but I do not attribute this advanced condition to the M. D. title, or believe that it is the essential factor to a high character of work. The title is the effect and not the cause, obtained for the reason that the possessor loves educational advancement. What I do believe, and what I would especially advocate as necessary to advanced thought, is a trained mind. It is the one thing essential.

If we desire to improve the character of our dental literature, it is to be done by raising the standard of dental education. The point at which to start is at the beginning—at the basis of admission of students to dental colleges. It ought to be required of those seeking the title of D. D. S. that they should give unquestionable evidence of a certain previously acquired educational advancement before they are admitted to enter upon the study of dentistry. Their minds should be disabused of the idea that they can study dentistry without much previous education, and that fullness, position and prominence, if desired, is to be had after the acquirement of D. D. S., by simply adding M. D.

To recapitulate: It has been shown that the character of our dental literature has improved in its scientific character in the last seven years.

That the subjects treated are not excessively practical, and the topic most considered during that time is not one fraught with the greatest good to the greatest number.

That the bulk of writing is done by graduates of dental colleges only.

That the number of active workers—doing most of such work—is exceedingly small, and out of all proportion to the large number engaged in dental practice.

And that the road to improvement lies in admitting men imbued at the outset with a desire for educational advancement.

In conclusion, I trust that those who have been reading with the blissful feeling that they were progressing will reflect upon the points I have mentioned, and will remember that if our journals do not bring us new facts in the future, the fault is not with the editors, but with ourselves.

